# Diet-induced metabolic changes of the human gut microbiome: importance of short-chain fatty acids, methylamines and indoles

**DOI:** 10.1007/s00592-019-01312-x

**Published:** 2019-03-22

**Authors:** Mohd Badrin Hanizam Abdul Rahim, Julien Chilloux, Laura Martinez-Gili, Ana L. Neves, Antonis Myridakis, Nigel Gooderham, Marc-Emmanuel Dumas

**Affiliations:** 1 Division of Systems and Digestive Medicine, Department of Surgery and Cancer, Imperial College London, Exhibition Road, London, SW7 2AZ UK; 2 Department of Biochemistry, Faculty of Biotechnology and Biomolecular Sciences, Universiti Putra Malaysia, 43400 UPM Serdang, Selangor Malaysia

**Keywords:** Gut microbiome, Short-chain fatty acids, Methylamine, Indoles, Microbial metabolism, G-protein-coupled receptor, Nuclear receptor

## Abstract

The human gut is a home for more than 100 trillion bacteria, far more than all other microbial populations resident on the body’s surface. The human gut microbiome is considered as a microbial organ symbiotically operating within the host. It is a collection of different cell lineages that are capable of communicating with each other and the host and has an ability to undergo self-replication for its repair and maintenance. As the gut microbiota is involved in many host processes including growth and development, an imbalance in its ecological composition may lead to disease and dysfunction in the human. Gut microbial degradation of nutrients produces bioactive metabolites that bind target receptors, activating signalling cascades, and modulating host metabolism. This review covers current findings on the nutritional and pharmacological roles of selective gut microbial metabolites, short-chain fatty acids, methylamines and indoles, as well as discussing nutritional interventions to modulate the microbiome.

## Introduction

The human intestinal tract provides the trillions of resident bacteria with a nutrient-rich environment and in exchange the host benefits as the gut microbiota helps process nutrients for our body needs. This symbiotic relationship is often described as mutualism, as both the bacteria and the host benefit from the interaction. *Bacteroides thetaiotaomicron*, is one of the most common and best-known microbes in human intestine, capable of degrading the indigestible dietary polysaccharides that serve the host with 10–15% of their calorific requirement [1]. A large number of microbial metabolic processes beneficial to the host are involved in the digestion and degradation of these indigestible dietary fibres. A number of gut microbiota species have been shown to be involved in the metabolism of dietary fibres to short-chain fatty acids (SCFAs), generating energy substrates for the host. Gut microbiota not only produce SCFAs but is also responsible for the production of other gut microbial metabolites such as methylamine from dietary choline, and indoles from the metabolism of aromatic amino acids like tryptophan [1].

In addition, resident microbes also contribute to the host’s fat homeostasis by modulating the uptake of dietary lipids. Gut microbiota regulate the storage of fat from calories harvested from the diet via acting through fasting-induced adipocyte factor (Fiaf). This increases hepatic lipogenesis via lipoprotein lipase (LPL) activity in adipocytes [1]. Conventionalisation of germ-free (GF) mice with normal mice cecal faecal microbiota, produces a 60% increase in total body fat content even with reduced food intake [2].

As the gut microbiota are involved in many processes during host growth and development, the imbalance in its composition and number may increase susceptibility to pathologies. Dietary intervention as well as prebiotic and probiotic treatments can alter microbial composition and improve bacterial gene richness [3]. Recently, a symbiotic which is a combination of probiotics and prebiotics, and probiotic supplementation has been shown to improve fasting plasma glucose, fasting insulin levels, as well as HbA1C in prediabetic subjects [4]. Moreover, the aberration in gut microbial community has also been shown to be associated with the development of gestational diabetes mellitus (GDM). Previously, a study reported that an increase in the relative abundance of the *Ruminococcaceae* family is positively correlated with the occurrence of GDM, with the possibilities that this bacterial family promotes inflammation-impaired glucose homeostasis leading to the reduction of insulin sensitivity [5]. Therefore, the balance of the microbiota species is important, as it can influence the health status of the host. The importance of this is seen in the host immune response as the gut microbiota are involved in the development of intestinal mucosa and systemic immune system throughout the life of the host [6]. Studies on germ-free (GF) animals revealed that gut microbiota play an important role in regulating physiological, biochemical, and immunological development of the host. GF animals have abnormal numbers of immune cell types and immune products. Furthermore, commensals are also involved in many important intestinal functions by modulating the gene expression profile of the intestinal epithelial cell layer [3]. Therefore, the presence of the gut microbial community is vital to the host, as it helps maintain gut health and resistance to pathogen colonisation [3].

Identifying the pharmacological targets and signalling properties of these gut microbial metabolites is vital for understanding the underlying mechanisms of the gut–microbial metabolites–host interaction in modulating host’s cellular functions. As these metabolites have been shown to interfere with host metabolism via several mechanisms, including acting as signalling molecules activating intracellular signalling cascades, we present three classes of gut microbial metabolites that play important roles in host molecular mechanisms: short-chain fatty acids (SCFA), methylamines, and indoles. We also discuss the strategies to manipulate the microbial ecology.

## Main

### Short-chain fatty acids

Consumption of dietary fibre has been epidemiologically associated with a lower incidence of metabolic diseases and cancers [7]. Fermentation of the dietary fibre (e.g., from cereal bran, fruit skins and seeds, vegetables and pulses, nuts) occurs predominantly in the proximal colon where substrate availability and bacterial activity are the highest. The fibre is converted into SCFA and other by-products of the microbial fermentation of carbohydrates including CO_2_, CH_4_, H_2_, bacterial cell mass, and heat [3, 6].

The main SCFAs produced are acetate, propionate, and butyrate; other SCFAs are also produced in much lower amounts, i.e., formate, valerate, caproate, and branched-chain fatty acids (BCFAs) [2, 3]. The SCFAs produced in this manner are released at high concentrations in the ascending colon (70–140 mM), and their concentration declines in the transverse colon (20–70 mM) and in the descending colon (20–40 mM) [3]. The molar ratio of acetate, propionate, and butyrate production in the colonic lumen is reported to be 60:25:15, respectively [2]. However, this ratio can change depending on several factors such as diet, microbial composition, and the site of fermentation [8].

**Fig. 1:**
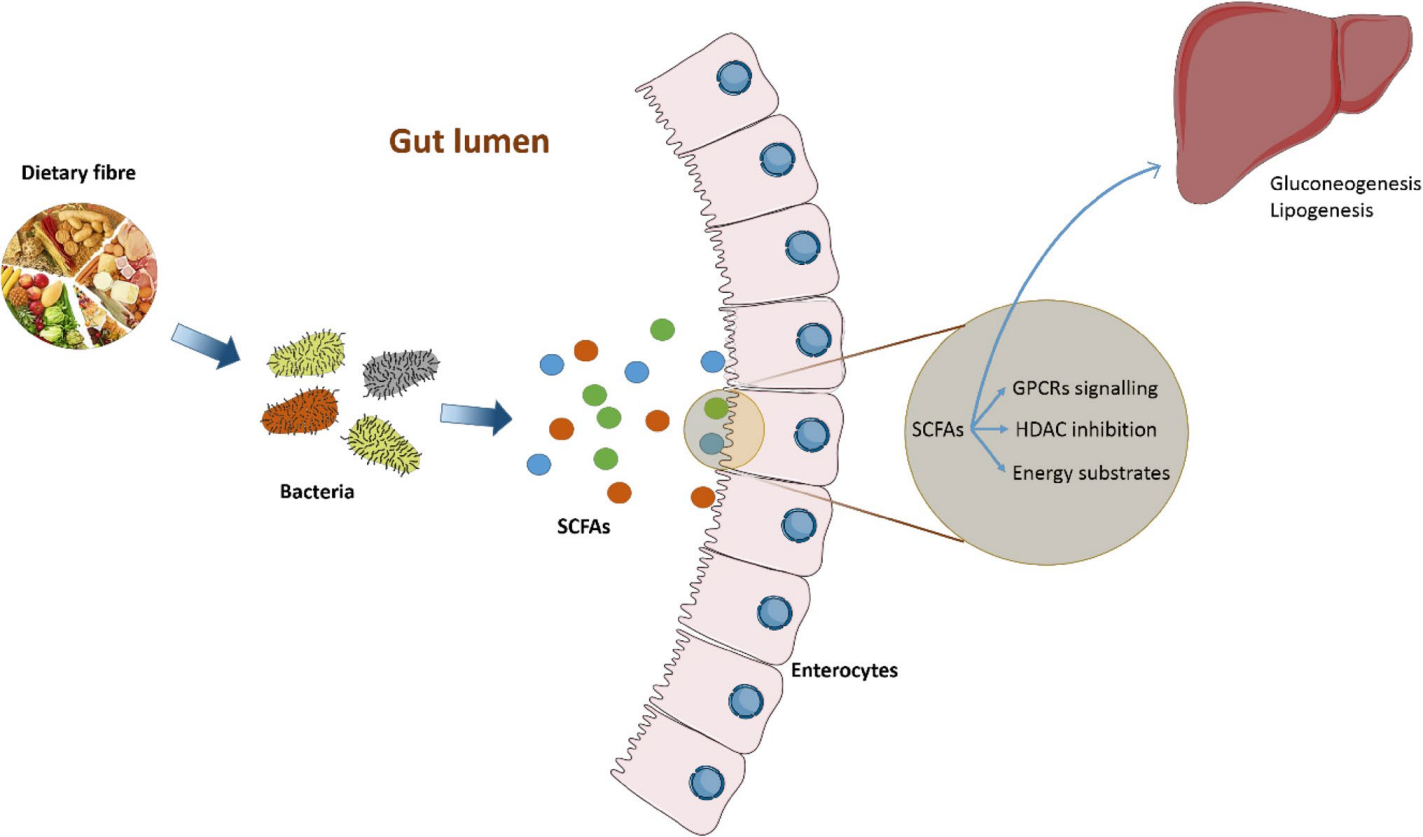
Roles of gut microbial metabolites (SCFAs) in human. Once absorbed in the colon, butyrate serves as energy substrates for colonocytes, and acetate and propionate are transported to the liver and peripheral organs. In addition, SCFAs can also act as HDAC inhibitor and regulate many physiological processes through signalling via GPCRs

One of the key properties of SCFAs is that they can act either as substrates for host metabolism and/or as signalling molecules (Fig. 1). Acetate, produced via the fermentation of carbohydrates by intestinal bacteria, is taken up by the gastrointestinal (GI) epithelium, released into the portal vein bloodstream to the liver, and eventually distributed to peripheral tissues where it is metabolised mostly by muscle [9]. Acetate can also cross the blood–brain barrier to activate acetyl-CoA carboxylase and expression of neuropeptides thereby inducing hypothalamic neuronal activation and suppressing appetite [10]. Moreover, acetate is the primary substrate for cholesterol synthesis, and may interfere directly in lipid metabolism [11]. High concentrations of acetate provide substrate for hepatic lipogenesis [12].

The liver clears the majority of propionate and butyrate from the portal circulation to prevent high SCFAs concentrations in blood [13]. Approximately, 50% of propionate is used by humans as a substrate for hepatic gluconeogenesis [14]. Propionate enters TCA cycle via the succinyl-CoA entry point. It is first converted into propionyl-CoA by propionyl-CoA synthetase, which is then converted into succinyl-CoA via three successive reactions. The resulting succinyl-CoA enters the TCA cycle and is converted into oxaloacetate, the gluconeogenesis precursor [15]. The concentrations of propionate in the portal vein versus the hepatic veins confirm the substantial uptake of propionate by the liver [16].

The effect of propionate on hepatic carbohydrate metabolism was supported by its role in improving glucose tolerance and insulin sensitivity, as well as increasing high-density lipoprotein (HDL) [17]. Moreover, it has been demonstrated that propionate is converted into glucose by intestinal gluconeogenesis (IGN), thus improving energy homeostasis [18].

Of the SCFAs produced by gut microbiota in human intestine, butyrate has caught the most attention and has been studied extensively, in particular supressing colonic inflammation, causing cell cycle arrest and apoptosis, highlighting its role in protecting against colon cancer and colitis [19, 20]. Butyrate is the principal substrate and energy source for colonocytes providing at least 60–70% of colonic mucosa energy requirements, essential for their proliferation and differentiation [21]. Inside the cell, butyrate enters mitochondria in which it undergoes β-oxidation to acetyl-CoA and enters the tricarboxylic acid cycle (TCA cycle) for energy production [22] which can prevent autophagy by rescuing the deficit in mitochondrial respiration and energy perturbation [23]. Butyrate is important in maintaining colonic epithelium formation via its role as an anti-inflammatory agent to prevent the production of reactive oxygen species and reactive nitrogen species generated in the event of oxidative stress [24]. Moreover, butyrate can also play a role in lipid metabolism, as well as exerting anti-tumorigenic effects on many cancer cell lines [24]. At least part of its beneficial effects is reported to be related to its ability to inhibit histone deacetylases (HDACs) [25].

Beyond their role as substrate for energy production, SCFAs also act as signalling molecules through cell surface receptors known as G-protein-coupled receptors (GPCRs).

FFAR2 and FFAR3 activation following ligand binding inhibits the production of cyclic adenosine monophosphate (cAMP)-dependent pathway by adenylate cyclase, resulting in the reduction of intracellular cAMP production from ATP via interaction with G_αi_ protein [26, 27]. The Gαq protein family activates phospholipase Cβ (PLCβ) isoforms to hydrolyse phosphatidylinositol 4,5 bisphosphate into 1,2 diacylglycerol (DAG) and inositol 1,4,5 triphosphate (IP3). While DAG acts as a second messenger that activates protein kinase C (PKC), IP3 binds to specific IP3 receptor calcium (Ca^2+^) release channels in the endoplasmic reticulum, thus increasing Ca^2+^ release [27].

A number of studies have demonstrated that FFAR2 acts as a chemoattractant receptor for SCFAs in neutrophils [27–29]. The expression of FFAR2 is frequently reduced or abolished in colon cancer cells; in fact restoration in the FFAR2 expression followed by propionate treatment induced G_0_/G_1_ cell cycle arrest and activated caspases, leading to apoptotic cell death [30]. Therefore, it is suggested that there is a possible link between the gut microbial fermentation products and FFAR2 in lowering colon cancer incidence [30]. FFAR2 is also found to be expressed in peptide YY (PYY)-expressing enteroendocrine cells (L cells) [31]. Additionally, it has been shown that SCFA triggers the production of glucagon like peptide (GLP-1), a gut hormone with anorexigenic properties, through FFAR2 [32, 33].

FFAR3 activation increases leptin secretion, a hormone that acts as a signal of satiety [34]. Butyrate and propionate induce intestinal gluconeogenesis, which has beneficial effects on glucose and energy homeostasis via two different mechanisms: the first by acting as FFAR3 agonist to induce intestinal gluconeogenesis gene expression and the second via gut–brain neural circuit involving the FFAR3 [18].

Butyrate was also identified as a ligand for HCAR2, whose activation promotes anti-inflammatory responses [35] and suppresses colonic inflammation and carcinogenesis [36]. More recently, it has been reported that the beneficial effects of high-fibre diet involves the activation of GPR109A and FFAR2 in the gut epithelium, thereby promoting gut epithelium homeostasis via the inflammasome pathway [36].

### Methylamines

Other microbiota-derived metabolites have been associated with metabolic disease. This is the case for methylamines such as trimethylamine (TMA) and trimethylamine N-oxide (TMAO).

TMA and TMAO were first associated with metabolic disorders through a study on insulin resistance and fatty liver disease [37]. TMAO was later on associated with atherosclerosis [38]. TMA typically results from bacterial metabolism of choline [39, 40], through choline:TMA lyase activity. l-Carnitine is another dietary substrate [41], converted to TMA through a microbial oxygenase [42]. This pathway is quite complex as γ-butyrobetaine, an intermediary substrate is also converted into TMA [43]. Finally, we recently demonstrated that the human gut microbiota retroconverts TMAO into TMA [44], following initial observations from Robert Smith et al. in the late 1980s [45].

TMA is absorbed and oxidised into TMAO by flavin-containing monooxygenase 3 (FMO3) during first-pass metabolism. Mutations in FMO3 cause trimethylaminuria, otherwise known as fish odour syndrome [46]. TMA also undergoes demethylation to form dimethylamine and monomethylamine.

Through its association with atherosclerosis, TMAO is mostly considered as proatherogenic, with a role in platelet hyperreactivity [47] and this has led to the development of inhibitors of the choline:TMA lyase, such as 3,3-dimethyl-1-butanol (DMB) and substituted analogues [48, 49]. TMA and TMAO are associated with metabolic improvements induced by *Akkermansia muciniphila* treatment in high-fat diet-fed mice [50]. TMAO also was associated with reduced endoplasmic reticulum (ER) stress, whilst chronic TMAO treatment in mice improves glucose tolerance and increases insulin secretion [51]. This is consistent with TMAO being an osmolyte, stabilising protein conformation and therefore counteracting ER stress generally observed in obesity and diabetes [52].

### Indoles

The gut bacterial ecosystem is also involved in the degradation of dietary aromatic amino acids (tryptophan, tyrosine, phenylalanine and histidine).

Tryptophan bacterial metabolism, in particular, has been extensively studied. Tryptophan is an essential amino acid, particularly abundant in cheese, poultry, red meat, egg white and seeds [53]. Tryptophanase is a lyase present in many bacterial species (e.g., *Bacteroides thethaiotamicron, Proteus vulgaris* and *Escherichia coli*) [54]; it directly catalyses the conversion of tryptophan to indole, which is further sulphated in the liver into 3-indoxylsulphate [55]. Although tryptophanase is the most studied enzyme, it represents only a small part of the complex network of bacterial reactions involved in the bacterial degradation of tryptophan. Tryptophan can also undergo deamination by *Clostridium* and *Lactobacillus* spp., producing a range of other indole-containing molecules (indole-3-lactate, indole-3-acetate and 3-methylindole) [56, 57]. Metabonomic studies demonstrated that the production of indoles depends heavily on bacterial activity: in an MS-based study comparing plasma extracts from conventional and germ-free mice, 3-indoxylsulphate and indole-3-propionate were present only in the serum of conventional mice [57].

Indoles were shown to impact several homeostatic processes relevant to its mammalian host (e.g., inflammation [58], gut barrier permeability [59]). Indoles exhibit affinity for the aryl hydrocarbon receptor (AhR), which has recognised functions in innate immunity and xenobiotic responses [55]. Of note, the effect is not homogenous: while indole-3-acetate is suggested to be a weak AhR agonist and partial antagonist [60], indole itself showed antagonist activity [61].

Indole-3-propionate is a pregnane X receptor (PXR) ligand in synergy with indole, which promotes the maintenance of the intestinal barrier integrity. As high-fat diets are known to increase intestinal permeability, bacterial translocation and increase inflammation, it is significant that IPA can promote beneficial effects on the host’s metabolism [62].

### Impact of microbial metabolites on behaviour

The connection between microbiota and brain has been an unexplored field until recently. There is increasing evidence that gut microbes participate in a myriad of neurological processes, from neurodevelopment, behaviour and ageing to neurodegenerative diseases [63].

Interestingly, there is a co-morbidity of neurological pathologies with metabolic and intestinal disorders. For example, numerous studies show a reciprocal relationship between anxiety or depression and obesity [64, 65]. Significantly, obese patients have a different microbiota composition and increased intestinal permeability and inflammation [66]. But is there a causal link between the gut microbiota and behavioural changes in obese patients? 4-Ethylphenylsulfate (4EPS), is a microbial metabolite derived from the intestinal fermentation of tyrosine, which is converted into 4-ethylphenol and subsequently sulfated in the liver. Increased levels of 4-ethylphenol have been found in urine of rats that are more prone to develop insulin resistance and obesity using drug and dietary insults [67]. Intriguingly, mice treated with 4EPS display anxiety-like behaviours [68]. Although these models suggest that a microbial metabolite could be related to both metabolic and neuronal perturbations, further metagenomic, metabolomic and behavioural studies in obese patients will be required to confirm this association.

A recent study comparing metabolically healthy obese patients (i.e., absence of inflammation and normal blood pressure, insulin sensitivity and lipid profiles), with unhealthy ones, found that the latter were more prone to suffer depression and anxiety [69]. Moreover, an intensive lifestyle improvement program has been shown to reduce the glycemic and lipid control as well as weight, along with significant changes in adipokines, cytokines and gut hormones levels even after 1 year post-intervention [70]. A full characterisation of the metabolome and metagenome of these patients might give more insight into the causality of these differences.

SCFAs are able to cross the blood–brain barrier (BBB) [71] and have been shown to be related with satiety. Particularly, acetate and propionate are able to suppress appetite through GPCR and neuronal signalling pathways [10, 72]. This implies that increasing fibre ingestion might have a synergistic positive effect on obesity, by reducing daily caloric intake and promoting satiety thanks to the increase in microbial-derived SCFA. At the same time, SCFAs are able to induce serotonin (5-HT) synthesis by enterochromaffin cells in the intestine [73]. Serotonin is involved in gut motility and has been also shown to have a feeding–suppressing action [74]. Intestinal serotonin is not capable of crossing the blood–brain barrier, however, its precursor tryptophan is. Tryptophan can also be synthesized by the intestinal microbiota, which can indirectly influence serotonin levels in the brain [75]. Finally, serotonin can also be involved in motor activity and circadian rhythm, which are important players in metabolic disorders.

### The importance of metabolomics to characterise the functional effects of the microbiome

The relationships between human gut microbiome composition, metabolism and disease risk are already established [76–78]. Therefore, metabolomics, the systematic study of the unique metabolic profile of a cell, tissue, organ or organism, can play a key role in gut microbiome research [79]. The most widely used techniques for metabolic profiling are proton nuclear magnetic resonance (^1^H NMR) spectroscopy [80] and mass spectrometry (MS) [81, 82]. ^1^H NMR produces reproducible and robust metabolomic data in biofluids (urine, cell media, blood, etc.) and requires minimal sample preparation. On the other hand, MS is more sensitive and capable of detecting metabolites at much lower levels and to improve its resolution, MS is usually coupled with either liquid (LC) or gas (GC) chromatography [83].

Several studies have associated many gut microbiome metabolite levels and health outcomes. Methylamines (trimethylamine, trimethylamine-N-oxide etc.) have been linked with progressive renal fibrosis/dysfunction [84], insulin resistance [37] and non-alcoholic fatty liver disease [83], atherosclerosis [41] and even in the experimental treatment of type 2 diabetes with *A. muciniphila* [50]. Moreover, branched-chain amino acids (BCCAs), membrane phospholipids and triacylglycerols have been connected with insulin resistance. Furthermore, short-chain fatty acids (SCFAs) and aromatic amino acids (AAAs), which are produced by bacterial fermentation of carbohydrates and proteins, also influence the host’s health [85]: in a recent study on the role of the microbiome in hepatic steatosis, phenylacetic acid, a microbial product of phenylalanine, was associated with steatosis and increased lipid storage in human primary hepatocytes and in mice [86].

## Conclusion

Increasing evidence identifies a determinant role of gut microbiota in the host’s health, and one underlying mechanism is via gut–microbial metabolites–host interaction in modulating host’s cellular functions. As these microbial communities are dynamic and can influence many physiological functions in our body, modifications in this gut–microbial ecology by external stimuli such as dietary factors, antibiotics, as well as pro- and prebiotics will contribute to host health and disease state. In particular, some metabolic disorders of the host have been associated with an inflammation-related environment caused by the imbalance of the specific gut bacterial strains. In view of the potential role of dietary fibre-derived SCFA on human health benefits, many studies from in vitro cell culture studies to animal model studies to human subjects have been conducted. The recent report that colonic delivery of SCFA has been associated with improved beta-cell function and insulin secretion [87], illustrating this association therefore signifies promising therapeutic avenue of the gut–microbial metabolites. Despite the increasingly strong evidence supporting the significant contribution of the gut–microbial community and its metabolites in host health and disease, more studies are needed to unravel the missing links between the gut–microbial and host metabolic axis to understand and improve human health in relation to metabolic disorders.
